# Evidence for heterogeneous nuclear ribonucleoprotein K overexpression in oral squamous cell carcinoma

**DOI:** 10.1038/sj.bjc.6603911

**Published:** 2007-07-31

**Authors:** P Roychoudhury, K Chaudhuri

**Affiliations:** 1 Molecular and Human Genetics Division, Indian Institute of Chemical Biology, 4 Raja SC Mullick Road, Kolkata 700032, India

**Sir**,

The recent paper of Carpenter *et al* (2006b) in your journal identified hnRNP K as being overexpressed in colorectal cancer by proteomics, which has been confirmed by immunohistochemistry and tissue microarray analysis. Their study also showed that hnRNP K had an aberrant subcellular localisation in cancer cells. Although there were previous reports by Moumen *et al* (2005), suggesting that, in response to DNA damage, p53 inhibits hnRNP K ubiquitin-dependent proteasomal degradation, the study by Carpenter *et al* (2006a) could not, however, find any correlation between the expression of hnRNP K and p53 in colorectal cancer cells. We note the above observations with interest, as hnRNP K has a role in tumorigenesis and its expression has been found to be upregulated in several other cancers, including those of lungs and liver (Carpenter *et al*, 2006a). Our pilot study with 26 oral cancer patients has also recorded the significant upregulation (*P*<0.001) of hnRNP K mRNA compared with normal oral tissue specimens using quantitative real-time RT–PCR (Figure 1A). The ΔCT of hnRNP K mRNA in cancer tissues (mean±s.d., 2.6±3.8) was significantly (*P*<0.001) lower than that in the corresponding normal tissues (mean±s.d., 5.48±2.3). As such, the increase in the expression of hnRNP K was about 56-fold in oral SCC compared to the normal oral epithelium. Among the 26 oral SCC patients, 11, 12 and 3 patients were examined as having well, moderately and poorly differentiated SCC, respectively. The average ΔCT value for well-differentiated SCC group was 3.26±3.6, whereas for moderately and poorly differentiated group is 2.12±4.05, that is, the expression of hnRNP K in well-differentiated SCC group was lower than the moderate and poorly differentiated SCC group (Figure 1B), but significant correlation between histological grades of differentiation and hnRNP K mRNA expression could not be predicted, a larger sample size is needed to decide upon true correlation. Furthermore, to investigate the correlation between hnRNP K and p53 in oral cancer, full cDNA of p53 was transiently transfected in an oral cancer cell line. The level of p53 achieved by transient transfection was significantly higher than its endogenous level in mock-transfected cells. Increased expression of p53 in transfected cells did not, however, alter the expression of hnRNP K, which therefore corroborate with the data presented by Carpenter *et al* (2006b) for colorectal cancer cells.

We hope subsequent studies with immunohistochemistry and western blotting on normal and oral cancer tissues with larger sample sizes will strengthen and validate our understanding the role of hnRNP K in cancer.

## Figures and Tables

**Figure 1:**
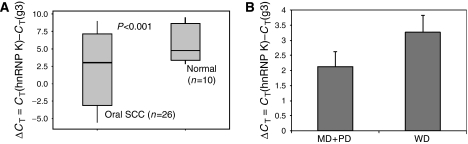
(**A**) Wide box representation of hnRNP K mRNA expression in cancerous and noncancerous oral tissues. Cancer tissues showed significantly lower level of ΔCT or higher level of hnRNP K mRNA expression levels compared to noncancerous tissues (*P*<0.001). (**B**) Relative level of hnRNP K mRNA expression in different histological grades of oral SCC with respect to normal oral tissue. W.D, M.D-P.D represent well, moderately to poorly differentiated oral SCC tissues. Values are means and s.d. from *n*=15 (M.D+P.D) and *n*=11(W.D) samples.
